# Molecular Detection of *Orthohantavirus puumalaense* in Plasma and Urine Samples from Hospitalized Patients Presenting with a Serologically Confirmed Acute Hantavirus Infection in France

**DOI:** 10.1128/jcm.00372-23

**Published:** 2023-07-24

**Authors:** Jean-Marc Reynes, Laura Schaeffer, Pavlos Papadopoulos, Mohand Ait-Ahmed, Dieyenaba Siby-Diakite, Maryline Ripaux-Lefèvre, Tan-Phuc Buivan, Sylvie Lechat, Muriel Vray, Jean-Marc Galempoix, for The HANTADIAG Study Group

**Affiliations:** a Institut Pasteur, Université Paris Cité, Unité Environnement et Risques Infectieux, Centre National de Référence Hantavirus, Paris, France; b Institut Pasteur, Université Paris Cité, Unité Epidémiologie des Maladies Emergentes, Paris, France; c Institut Pasteur, Université Paris Cité, Centre de Recherche Translationnelle, Paris, France; d Centre Hospitalier Intercommunal nord Ardennes, Laboratoire de Biologie Médicale, Charleville- Mézières, France; e Centre Hospitalier Intercommunal nord Ardennes, Service de Médecine Interne et Maladies Infectieuses, Charleville- Mézières, France; Institut Pasteur, Université Paris Cité, Unité Environnement et Risques Infectieux, Centre National de Référence Hantavirus, Paris, France.; Institut Pasteur, Université Paris Cité, Unité Environnement et Risques Infectieux, Centre National de Référence Hantavirus, Paris, France.; Institut Pasteur, Université Paris Cité, Unité Environnement et Risques Infectieux, Centre National de Référence Hantavirus, Paris, France.; Institut Pasteur, Université Paris Cité, Unité Environnement et Risques Infectieux, Centre National de Référence Hantavirus, Paris, France.; Institut Pasteur, Université Paris Cité, Unité Epidémiologie des Maladies Emergentes, Paris, France.; Institut Pasteur, Université Paris Cité, Centre de Recherche Translationnelle, Paris, France.; Institut Pasteur, Université Paris Cité, Unité Epidémiologie des Maladies Emergentes, Paris, France.; Institut Pasteur, Université Paris Cité, Centre de Recherche Translationnelle, Paris, France.; Centre Hospitalier Intercommunal nord Ardennes, Service de Médecine Interne et Maladies Infectieuses, Charleville- Mézières, France.; Centre Hospitalier Intercommunal nord Ardennes, Laboratoire de Biologie Médicale, Charleville- Mézières, France.; Institut Pasteur, Université Paris Cité, Unité Environnement et Risques Infectieux, Centre National de Référence Hantavirus, Paris, France.; Centre Hospitalier Intercommunal nord Ardennes, Service de Néphrologie, Charleville- Mézières, France.; Hôpital Nord Franche-Comté, Service d’Infectiologie, Trévenans, France.; Hôpital Nord Franche-Comté, Laboratoire de Biologie Médicale, Trévenans, France.; Centre Hospitalier Universitaire de Besançon, Service des Maladies Infectieuses et Tropicales, Besançon, France.; Centre Hospitalier Universitaire de Besançon, Laboratoire de Virologie, Besançon, France.; Centre Hospitalier Universitaire Dijon-Bourgogne, Département d’Infectiologie, Dijon, France.; Centre Hospitalier Universitaire Dijon-Bourgogne, Laboratoire de Sérologie-Virologie, Dijon, France.; Centre Hospitalier de Laon, Service de Médecine Interne - Maladies Infectieuses, Laon, France.; Centre Hospitalier de Laon, Service des Urgences, Laon, France.; Centre Hospitalier de Laon, Laboratoire d’Analyses Médicales - Hygiène Hospitalière, Laon, France.; CHRU-Nancy, Université de Lorraine, Service Maladies Infectieuses et Tropicales, Nancy, France; Université de Lorraine, APEMAC, Nancy, France.; CHRU-Nancy, Université de Lorraine, Département de Microbiologie, Laboratoire de Virologie, Nancy, France.; Centre Hospitalier Universitaire de Reims, Service de Médecine Interne, Maladies Infectieuses et Immunologie Clinique, Reims, France.; Centre Hospitalier Universitaire de Reims, Service de Néphrologie, Reims, France.; Centre Hospitalier Universitaire de Reims, Laboratoire de Bactériologie-Virologie-Hygiène-Parasitologie-Mycologie, Reims, France.; Centre Hospitalier Louis Jaillon, Service de Médecine, Saint-Claude, France.; Centre Hospitalier de Lons-le Saunier, Laboratoire de Biologie Médicale, Lons-le-Saunier, France.; Centre Hospitalier de Verdun Saint-Mihiel, Service territorial Dialyse et Néphrologie, Verdun, France.; Centre Hospitalier de Verdun Saint-Mihiel, Laboratoire de Biologie Médicale, Verdun, France.; Cepheid

**Keywords:** France, molecular diagnostic, nephropathia epidemica, orthohantavirus, plasma, Puumala hantavirus, urine, hemorrhagic fever with renal syndrome

## Abstract

Molecular detection of Orthohantavirus puumalaense (PUUV) RNA during the course of the disease has been studied in blood of patients in Sweden and Slovenia. The use of urine has been poorly investigated. The aims of this work were to study PUUV RNA detection in plasma from a cohort of patients in France where a different PUUV lineage circulates and to assess the use of urine instead of plasma. Matched plasma and urine samples were collected daily from hospitalized patients presenting with fever, pain, and thrombocytopenia within the last 8 days and testing positive for IgM and IgG against PUUV in serum collected at inclusion and/or approximately 1 month after release. RNA was extracted from samples, and PUUV RNA was detected using real-time reverse transcription-PCR for plasma and urine samples. Sixty-seven patients presented a serologically confirmed acute hantavirus infection. At inclusion, PUUV RNA was detected in plasma from 55 of 62 patients (88.7%) sampled within the first week after disease onset, whereas it was detected in 15 of 60 (25.0%) of matched urine samples. It was then detected from 33 (71.7%) and 2 (4.4%) of 46 patients discharged from the hospital during the second week after disease onset, in plasma and urine, respectively. When PUUV RNA was detected in urine it was also detected in plasma, and not vice versa. Detection of PUUV RNA in plasma from hospitalized patients in France is similar to that observed in Sweden and Slovenia. Urine is not an appropriate sample for this detection.

## INTRODUCTION

Hantaviruses are enveloped viruses containing a negative-sense single-stranded trisegmented RNA genome and grouped within the family *Hantaviridae* (order *Bunyavirales*). Their natural hosts include rodents, insectivorous small terrestrial mammals (moles and shrews), and bats, but also fishes and one reptile (1). Some of the hantaviruses associated with the rodents are responsible for a severe hantavirus pulmonary syndrome (HPS) or a mild, moderate, or severe hemorrhagic fever with renal syndrome (HFRS) in human. *Orthohantavirus puumalaense* (PUUV), responsible for a mild HFRS called nephropathia epidemica, is the most prevalent species detected in patients in Europe, with thousands of cases reported per year (2). PUUV is also the main hantavirus detected in humans in metropolitan France, while *Orthohantavirus seoulense* (SEOV) is sporadically detected and *Orthohantavirus tulaense* was detected only once (3). Today, eight PUUV geographically structured lineages are described, and the PUUV strains circulating in France have been shown to belong to the Central European lineage (4).

Since the discovery of hantaviruses in the 1980’s and later, hantavirus laboratory diagnostics have been based on serological assays; molecular techniques are principally used to detect and sequence hantavirus strains for molecular phylogeny and epidemiology (5). However, the acquisition of sequences has allowed the improvement of molecular assays and their use in diagnostics (2). In this way, PUUV has been detected in serum or plasma during at least the febrile phase of the disease. The PUUV plasma load and its kinetics have been studied in Sweden and Slovenia, where the PUUV Scandinavian lineages and the Alpes Adrian lineage are reported, respectively (6–9).

Alternative noninvasive samples, e.g., urine and saliva, have been used with more or less success for the diagnosis of emerging infectious viral diseases (10). Although most zoonotic hantaviruses usually cause renal dysfunction directly or indirectly in humans, the use of urine for the molecular diagnosis of hantavirus infections is uncommon (11–14) and has been rarely reported for PUUV diagnosis, with various degrees of success in small cohorts of patients (15–18).

In this way, the objective of this work was to assess the presence of PUUV in plasma and urine from a large cohort of hospitalized patients in France presenting with a serologically confirmed acute hantavirus infection.

## MATERIALS AND METHODS

### Study sites.

Study sites were 9 public hospitals (Belfort-Montbéliard, Besançon, Charleville-Mézières, Dijon, Laon, Nancy, Reims, Saint-Claude, and Verdun) located in the northeastern part of metropolitan France, where the French PUUV is endemic.

### Puumala virus suspected clinical cases.

Suspected cases were hospitalized patients, male or female, between 18 and 75 years old who (i) had pain and febrile syndrome (body temperature, ≥38°C) at inclusion, (ii) had a platelet count of ≤150 g/liter within the last 8 days, (iii) were living in the area where PUUV is endemic, and (iv) gave their written consent after being informed of the research and the collection of data as well as blood and urine samples.

The following patients were excluded: (i) those known to have been previously infected by a hantavirus (based on medical records and/or laboratory results); (ii) those known to present stable thrombocytopenia; (iii) those who would not adhere to the protocol according to the medical staff, or (iv) those whose health status may interfere with the study or was not compatible with the sampling planned in the study.

### Clinical samples.

Serum samples were obtained from blood (10 mL) collected from suspected cases at inclusion and about 1 month after hospital discharge. Serum aliquots were stored at −20°C until testing.

Urine (5 mL) and blood in EDTA tube (5 mL) samples were collected at inclusion from all suspected cases and collected daily during hospitalization in those patients who tested positive for IgM anti-Puumala virus using the rapid test POC Puumala IgM or ReaScan+ Puumala IgM from Reagena (the rapid test was used as a first-line serological test to avoid unnecessary daily sampling of hantavirus-noninfected patients). Urine and plasma aliquots were stored at −80°C until testing.

### Hantavirus serological diagnosis.

Collected sera were screened at a dilution of 1:100 for the presence of anti-PUUV and Thailand virus (THAIV; representative of Seoul virus) IgG and IgM using a home-made enzyme-linked immunosorbent assay (ELISA) performed according to Rossi et al.’s method (19). Positive results obtained by ELISA were confirmed using a home-made immunofluorescence assay according to Niklasson et al.’s technique (20); sera were tested at a dilution of 1:64 against PUUV virus-infected cells and THAIV-infected cells. Both assays were conducted under ISO 15189 accreditation. A hantavirus acute infection was defined by the detection of IgM and IgG against hantavirus, a hantavirus past infection by the detection of only IgG (and not IgM) against hantavirus, and a lack of hantavirus infection by the absence of IgG seroconversion (negative to positive), while an undetermined status was declared in other situations (e.g., absence of IgG against hantavirus at the inclusion but lack of the convalescent-phase serum to look for a seroconversion).

### PUUV molecular diagnosis.

A volume of 100 μL of plasma or urine plus 40 μL of DNase/RNase-free water was first spiked with 10 μL of a known amount of a Sigmavirus from *Drosophila* (used as an internal control of RNA extraction), and then RNA was extracted from the 150-μL mixture using the NucleoSpin Dx virus kit (Macherey-Nagel, Germany). Extracted RNA quality was finally assessed by the partial amplification of the Sigmavirus genome using a real-time reverse transcription-PCR (RT-PCR) method (21). RNA extraction was validated if the cycle threshold (*C_T_*) value was in the expected range. Otherwise, the extraction was repeated once and then considered validated or not.

PUUV RNA detection was carried out in duplicate according to a real-time RT-PCR performed according to Kramski et al.’s method (22). Positive results were qualitatively accepted even if the RNA extraction was not validated, while negative results were considered undetermined when the extraction was not validated. Absolute quantification was achieved using a standard curve established with the European Virus Archive Global project, reference PUUV RNA (Ref-SKU 007N-EVA370). A total of 10^1^ RNA copies/assay mixture, corresponding to 2 × 10^3^ equivalent RNA copies/mL of sample was the last point detected in 100% of the assays.

Both assays were conducted under ISO 15189 accreditation.

### Clinical and biological data.

Comorbidity at inclusion, daily clinical signs, and biological parameters, including platelet and leukocyte counts, plasma creatinine level, C-reactive protein concentration, and proteinuria were collected, when available, from the medical records during hospitalization. A severe acute kidney injury (AKI) was defined by a plasma creatinine level of >353.6 μmol/liter (23).

### Statistical analysis.

To describe the characteristics of patients at inclusion, median (with interquartile range) and percentages were reported for continuous and categorical variables, respectively.

The proportion of patients with PUUV RNA detected in plasma and its confidence interval (CI), at the 95% level, were calculated at inclusion and at discharge according to the number of days after disease onset and then during the entire follow-up. The same analysis was done with the urine samples.

Statistical analyses were performed using Stata version 13 software (StataCorp LP, College Station, TX).

### Ethics statement.

This study (HANTADIAG) was registered with ClinicalTrials.gov (NCT02455375) and received ethical approval by the Comité de Protection des Personnes EST III. Written informed consent was obtained from all participants.

## RESULTS

**FIG 1:**
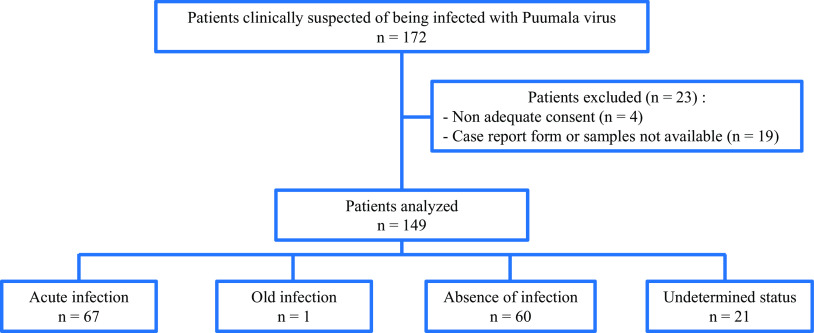
Flow diagram of patients enrolled in the HANTADIAG study.

Among the 172 PUUV patients with suspected clinical cases who participated in the study from July 2015 through December 2021, 23 were removed because of no adequate consent, lack of case report form, or lack of samples. Data and samples were analyzed for 149 cases, and finally 67 presented a serologically confirmed acute infection by a hantavirus (Fig. 1). Among them, 47 had IgM and IgG against hantavirus in the serum collected at inclusion and 20 had evidence of IgG antihantavirus seroconversion (IgM being present at least in the serum collected at inclusion).

Characteristics of these 67 patients are detailed in Table 1. They were mostly men (86.6%), and the median age was 37 years. Twenty-eight of 65 cases (43.1%; 2 were missing data) presented with HFRS (plasma creatinine of >120 μmol/liter) at inclusion, and 40/62 (64.5%; 5 were missing data) when including follow-up. A severe AKI was observed at inclusion for 7/65 cases (10.8%; 2 were missing data) and for 13/56 cases (23.2%; 11 were missing data) when including follow-up.

**TABLE 1:** Characteristics at inclusion of the 67 hospitalized patients presenting with a serologically confirmed acute hantavirus infection, France, 2015 to 2021

Characteristic	Value
No. (%) of males/total	58/67 (86.6%)
Median age, yrs (interquartile range [IQR])	37 (32–49)
Interval between symptomatic onset and inclusion (days) [IQR]	5 (3–7)
Interval between admission and inclusion (days) [IQR]	2 (1–2)
Duration of follow-up (days) at hospital [IQR]	5 (4–7)
No. (%) with:	
Fever (≥38°C)	37/67 (44.8%)
Headache	50/66 (75.8%)
Myalgia	44/67 (65.7%)
Backache	38/66 (57.6%)
Abdominal pains	31/67 (46.3%)
Nausea, vomiting	30/67 (44.8%)
Diarrhea	7/61 (11.5%)
Hepato-splenomegaly	7/67 (10.4%)
Visual disorder	23/67 (34.3%)
Cough	21/67 (31.3%)
Dyspnea	4/67 (6.0%)
Neurological disorder (meningitis signs)	1/67 (1.5%)
Hemorrhage requiring transfusion	0/67 (0%)
Hypertension	8/66 (12.1%)
Hypotension	2/66 (3.0%)
Shock	0/67 (0%)
Platelet count, g/liter (IQR; no. with missing data)	94 (68–142; 2)
Plasma creatinine, μmol/liter (IQR; no. with missing data)	104 (75–264; 2)
No. (%) with plasma creatinine level of >120 μmol/liter	28/65 (43.1%)
No. (%) with plasma creatinine level of >353.6 μmol/liter	7/65 (10.8%)

### PUUV RNA in plasma.

At inclusion, PUUV RNA was detected in plasma of 59 of the 67 patients (88.1%, 95% CI 77.8 to 94.7). Considering the number of days between the disease onset and sampling, PUUV RNA was identified in plasma from the 18 patients (100%, 95% CI 81.5 to 100) sampled until three days after onset, from 37 of the 44 patients (84.1%, 95% CI 69.9 to 93.4) sampled 4 to 7 days after onset and from 55 of the 62 patients (88.7%; 95% CI 78.1 to 95.3) sampled within the first week after onset. The median viral load was at a maximum 2 days after onset (1.43 × 10^5^ PUUV RNA copies/mL) and then decreased (minimum of 4.08 × 10^3^ PUUV RNA copies/mL) 6 days after onset (Table 2).

**TABLE 2:** Puumala virus molecular detection using real-time RT-PCR and viral load in plasma collected from the 67 hospitalized patients presenting with a serologically confirmed acute hantavirus infection, France, 2015 to 2021

Day post-disease onset	Patient no.	RT-PCR result			PUUV RNA load (copies/mL)	
		Indeterminate^*a*^	Negative	Positive	Extraction validated^*b*^	Median (range)
At inclusion						
2	7	0	0	7	6	1.43 × 10^5^ (9.13 × 10^3^–3.59 × 10^5^)
3	11	0	0	11	11	9.79 × 10^4^ (1.26 × 10^4^–8.76 × 10^5^)
4	10	0	1	9	7	1.99 × 10^4^ (2.32 × 10^3^–4.45 × 10^6^)
5	12	0	1	11	10	4.02 × 10^4^ (1.09 × 10^3^–5.81 × 10^5^)
6	8	0	1	7	6	4.08 × 10^3^ (5.55 × 10^2^–9.01 × 10^4^)
7	14	3	1	10	9	1.49 × 10^4^ (1.85 × 10^2^–2.71 × 10^5^)
8	4	0	1	3	3	3.54 × 10^3^ (2.45 × 10^3^–5.68 × 10^4^)
9	1	0	0	1	1	1.14 × 10^3^
Total	67	3	5	59	53	
At discharge						
3	1	0	0	1	1	7.24 × 10^4^
5	1	0	0	1	1	5.81 × 10^5^
6	7	0	1	6	6	1.51 × 10^4^ (5.4 × 10^2^–4.15 × 10^4^)
7	7	1	1	5	5	1.24 × 10^3^ (5.16 × 10^2^–1.41 × 10^4^)
8	10	0	2	8	8	2.6 × 10^3^ (1.99 × 10^2^–2.43 × 10^4^)
9	14	1	1	12	12	3.05 × 10^3^ (8.21 × 10^2^–6.56 × 10^4^)
10	8	2	1	5	3	1.33 × 10^3^ (1 × 10^0^–6.46 × 10^3^)
11	8	1	3	4	3	1.28 × 10^3^ (1 × 10^0^–6.20 × 10^3^)
12	2	0	0	2	2	2.26 × 10^3^ (1.94 × 10^3^–2.57 × 10^3^)
13	2	0	0	2	1	4.18 × 10^3^
14	2	1	1	0	0	
Total	62	6	10	46	42	

a The result of the real-time RT-PCR was negative but the extraction was not validated (late detection of the internal positive control).

b Quantification of the RNA load was considered positive for samples when the RNA extraction was validated.

During the whole follow-up, PUUV RNA was detected in plasma from most (*n* = 64) of the 67 patients (95.5%; 95% CI 87.5 to 99.1). PUUV RNA was detected 1 day after inclusion, at a low viral load, in 5 of the 8 PUUV RNA-negative patients at inclusion. The 3 remaining PUUV RNA-negative patients during the whole course of follow-up were cases with plasma samples obtained 7 days (2 cases) or 8 days (1 case) after disease onset (see Table S1 in the supplemental material). Positive detection of PUUV RNA or a high viral load were not significantly associated with severe AKI.

At discharge, plasma samples were available from 62 of the 67 patients. PUUV RNA was detected in 46 of the 62 cases (74.2%; 95% CI 61.5 to 84.4). Considering the number of days between disease onset and sampling, PUUV RNA was identified in plasma from 13 of the 16 patients (81.25%; 95% CI 54.4 to 96.0) sampled within the first week after onset and still from the majority (*n* = 33) of the 46 patients (71.7%; 95 CI 56.5 to 84.0) leaving the hospital during the second week after onset. The median viral load was quite stable during this second week after onset, ranging from to 1.3 × 10^3^ to 1.1 × 10^4^ PUUV RNA copies/mL (Table 2).

### PUUV RNA in urine.

At inclusion, urine was available from 65 patients and PUUV RNA was detected in 16 of them (24.6%; 95% CI 14.8 to 36.9). Considering the number of days between disease onset and sampling, PUUV RNA was identified in urine from 4 of the 18 patients (22.2%; 95% CI 6.4 to 47.6) sampled until 3 days after onset, from 11 of the 42 patients (26.2%; 95% CI 13.9 to 42.0) sampled 4 to 7 days after onset, and finally from 15 of the 60 patients (25.0%; 95% CI 14.7 to 37.9) sampled within the first week after onset. The median loads were less than 10^4^ copies/mL whatever the day after onset (Table 3).

**TABLE 3:** Puumala virus molecular detection using real-time RT-PCR and viral load in urine collected from the 67 hospitalized patients presenting with a serologically confirmed acute hantavirus infection, France, 2015 to 2021

Day post-disease onset	Patient no.	RT-PCR result			PUUV RNA load (copies/mL)	
		Indeterminate^*a*^	Negative	Positive	Extraction validated^*b*^	Median (range)
At inclusion						
2	7	0	5	2	2	2.19 × 10^3^ (7.5 × 10^2^–3.62 × 10^3^)
3	11	0	9	2	2	3.26 × 10^3^ (7.5 × 10^2^–1.6 × 10^3^)
4	9	0	6	3	3	2.88 × 10^3^ (1.3 × 10^1^–5.71 × 10^3^)
5	12	0	8	4	4	1.13 × 10^3^ (6.24 × 10^2^–1.58 × 10^3^)
6	7	0	5	2	2	4.96 × 10^3^ (9.74 × 10^2^–8.94 × 10^3^)
7	14	0	12	2	2	6.76 × 10^2^ (1–1.35 × 10^3^)
8	4	0	3	1	1	1.94
9	1^*a*^	0	1	0	0	
Total	65	0	49	16	16	
At discharge						
3	1	0	1	0		
5	1	0	0	1	1	8.73 × 10^2^
6	7	0	7	0		
7	7	0	6	1	1	5.82 × 10^2^
8	10	0	9	1	1	1.94 × 10^3^
9	14	0	14	0		0
10	8	0	8	0	0	0
11	8	0	8	0	0	0
12	2	0	1	1	1	1.72 × 10^3^
13	2	0	2	0	0	0
14	2	0	2	0	0	0
Total	62	0	58	4	4	

a The result of the real-time RT-PCR was negative but the extraction was not validated (late detection of the internal positive control).

b Quantification of the RNA load was considered positive for samples when the RNA extraction was validated.

During the entire follow-up, urine was available from all 67 patients and PUUV RNA was detected from only 25 patients (37.3%; 95% CI 25.8 to 50.0%). When PUUV RNA was detected in urine it was also detected in plasma, and not vice versa. When PUUV RNA was detected in both urine and plasma collected at the same time, the viral load in plasma was always higher, except for 3 pairs of samples. In those samples, the values were similar and low (less than 6 × 10^2^ copies/mL) (Table S1). PUUV RNA detection in urine was associated with the presence of a severe AKI (72.7% [8/11] versus 35.9% [14/39]; *P* = 0.044; data were missing for 17 cases), while high viral load was not.

At discharge, among the 62 urine samples available from the 67 patients, PUUV RNA was detected in only 4 patients (6.5%; 95% CI 1.8 to 15.7). Considering the number of days between disease onset and sampling, PUUV RNA was identified in urine from 2 of the 16 patients (12.5%; 95% CI 1.6 to 38.4) sampled within the first week after onset and from 2 of the 46 patients (4.4%; 95 CI 0.5 to 14.8) leaving the hospital during the second week after onset. The viral loads were less than 2 × 10^3^ PUUV RNA copies/mL (Table 3).

## DISCUSSION

At inclusion, PUUV was detected in plasma from most (88.7%) of the 62 patients sampled within the first week after disease onset, showing that PUUV molecular detection in plasma is efficient as a diagnostic test during acute infection. It is complementary to the antibody detection, especially when IgM detection is solely used, which has been known for lacking specificity (24). Our results are also consistent with those from earlier studies with a similar limit of detection of PUUV RNA using real-time RT-PCR. Indeed, in Sweden, where Scandinavian lineages circulate, PUUV RNA was detected in sera from 98.7% of the 79 patients sampled within 8 days after disease onset (9) or in sera from 90% of the patient sera collected at days 0 to 3 after onset and in 81% of the patient sera collected at days 4 to 7 (8).

The design of our study based on hospitalized patients, without follow-up after hospital discharge, did not allow us to assess the exact duration of the viremia. However, PUUV was still detected in plasma from a significant majority (71.7%) of the 46 patients leaving the hospital during the second week after onset. Thus, the duration of viremia in France is in the same range as that observed in Sweden, where PUUV was detected in the sera from 46% of the 105 patients sampled during the second and third weeks of disease (8), and that observed in Slovenia, where the PUUV Alpes Adrian lineage is reported (mean duration of viremia of 16 days after onset) (7). Additionally, our results confirmed that molecular diagnosis is less suitable than serological diagnosis at this stage (second week after onset), with IgM and IgG against hantavirus being detected in 100% of the cases (25). However, PUUV RNA detection in such cases remains useful to identify the hantavirus species responsible of the infection and for genotyping. The mean viral load in the range of 10^5^ RNA copies/mL observed within the first week after onset was similar to those observed in Sweden and Slovenia (7, 8), indicating no difference for this item between strains from 3 different lineages.

Hantavirus replication has been shown in tubular epithelial cells, podocytes, and glomerular endothelial cells, causing renal structure damage through cell-to-cell contact rupture rather than cell death (26). Presence at least of the virus genome in urine could be expected. However, our results obtained in tens of patients indicated that urine is not a suitable sample for the molecular diagnosis of PUUV infection in hospitalized patients sampled within the first 2 weeks after onset. Surprisingly, detection of measles virus and some arbovirus RNA in urine has been shown useful, since better results were obtained in this noninvasive sample than in plasma in the late course of the disease, although these viruses are not well-known for their renal damage (27–31). The pathological mechanism leading to the presence or the absence of the RNA from all these viruses, including hantaviruses, remains to be elucidated.

To our knowledge, 2 other series of cases have reported consistent results of detection of PUUV RNA in matched urine and serum or plasma samples. Urine samples collected daily until 5 days after disease onset from a mild case infected by PUUV in Russia, reported by Vetter et al., tested negative, while the plasma tested positive (18). PUUV RNA detection was also negative in both samples collected at admission (2 to 6 days after onset) from 10 Swedish patients (15). All 36 urine samples collected from 6 severe hospitalized cases infected by *Orthohantavirus sinnombreense* (SNV) also tested negative, while most of the 36 matched plasma tested positive (limit of RNA detection, 5,000 copies/mL) (14). However, better results were obtained from patients infected by *Orthohantavirus hantanense* (HTNV). HTNV RNA was detected from both urine and plasma samples collected within the first week of the disease from 4 patients with mild or moderate HFRS but at a lower viral load in urine (limit of detection not indicated) (11). HTNV RNA detection showed discordant results between paired plasma (or blood) and urine samples in a series of 8 cases infected by HTNV. In particular, HTNV RNA was detected only in urine from 2 severe cases sampled within the first week of the disease, while the plasma collected on the same day tested negative (this unique occurrence was not observed in our study). However, HTNV RNA was detected from blood or plasma collected later from these 2 cases (13). Urine sediment obtained after centrifugation has been used for the detection of PUUV RNA. A Finnish team detected PUUV in the urine sediment of 5 among 6 patients sampled within 1 week after onset and with a better percentage of success than using peripheral blood mononuclear cells sampled at the same time (17), and a Swedish team detected PUUV RNA in the urine sediment of 4 patients with higher signals than those obtained from lymphocytes collected at the same time (16). However, PUUV RNA detection was not performed in comparison with the whole urine and plasma or serum in these 2 studies; therefore, a positive result for these specimens could not be excluded. Comparison was made in the series of 6 SNV cases reported above, but SNV RNA detection in urine sediment was no more successful than in urine (14). Our PUUV RNA detection was not improved either in urine sediments obtained after centrifugation (15,000 × *g* for 1 h) from the 6 urine samples from patient 01/100/002 (data not shown, but see Table S1). Altogether, these results suggested the use of sediment urine does not improve hantavirus RNA detection. An option to consider is the use of disposable ultrafiltration centrifugal devices, allowing the concentration of nucleic acids from urine. A human apolipoprotein H capture assay, which has been proven effective to concentrate Andes virus in urine from a few cases, may be another option to be tested to improve PUUV RNA detection from this noninvasive sample (12).

In conclusion, our results confirmed that PUUV RNA detection in plasma is complementary to serological assays in early diagnosis of PUUV infection. Duration of viremia in plasma is similar to that observed in Sweden and Slovenia, where different PUUV genotypes circulate. The use of urine for the molecular diagnosis of this infection is not indicated.
